# Associations between health literacy and information-evaluation and decision-making skills in Japanese adults

**DOI:** 10.1186/s12889-022-13892-5

**Published:** 2022-08-02

**Authors:** Kazuhiro Nakayama, Yuki Yonekura, Hitomi Danya, Kanako Hagiwara

**Affiliations:** Graduate School of Nursing Science, St. Luke’s International University, 10-1 Akashi-cho, Chuo-ku, Tokyo, 104-0044 Japan

**Keywords:** Health literacy, Health information, Decision-making process, Shared decision-making, Learning opportunities

## Abstract

**Background:**

Health literacy among Japanese is often low, making it difficult for them to evaluate health information and make informed decisions. However, the health literacy scales applied measure the perceived difficulty of health-related tasks; they do not directly assess the specific skills needed to perform the tasks: the skills to judge the reliability of diverse information using evaluation criteria and implement rational decision-making. Therefore, the study objectives were to investigate the following issues using a nationwide survey in Japan. (1) When obtaining information, to what extent do people apply criteria for evaluating information to confirm its reliability; when making decisions, to what extent do they seek out available options and compare pros and cons based on their own values? (2) How strongly are such skills associated with health literacy and demographic characteristics? (3) What opportunities are available to learn these skills?

**Methods:**

We conducted an online questionnaire survey using a Japanese Internet research company; 3,914 valid responses were received. The measures comprised health literacy (European Health Literacy Survey Questionnaire), five items on information evaluation, four items on decision-making, and items on the availability and location of learning opportunities. We calculated Pearson correlations to explore the association of health literacy with information-evaluation and decision-making skills. Multivariate analyses were also conducted using these factors as dependent variables.

**Results:**

Fewer than half (30%–50%) of respondents reported always or often evaluating information and engaging in decision-making. Health literacy was significantly and positively correlated with the specific skills of information evaluation and decision-making (*r* = .26 and .30, respectively) as were multivariate analyses (beta = .15 and .22, respectively).

Over 40% of respondents had never learned those skills. The most common resources for learning the skills were the Internet and television; less-used resources were schools and workplaces.

**Conclusions:**

Both information-evaluation and decision-making skills were associated with health literacy. However, these skills are not sufficiently widespread in Japan because there are few opportunities to acquire them. More research is needed to raise awareness of the importance of such skills for improving health literacy and providing learning opportunities.

**Supplementary Information:**

The online version contains supplementary material available at 10.1186/s12889-022-13892-5.

## Background

Health literacy is the ability to access, understand, appraise, and apply health information [1]. Appraisal is the ability to evaluate information, and application refers to the ability to make informed decisions. Health literacy can help people become more empowered in relation to health care, disease prevention, and health promotion. The European Health Literacy Survey Questionnaire [2] has been used around the world [3] and in Japan [4–12] to measure comprehensive health literacy. Health literacy in Japan is associated with the following: health status and health behaviors in the general population [4–7]; social activity, exercise habits, and quality of life in patients [8, 9]; and a lower risk of atherosclerosis and lower frailty in elderly people [10–12].

In 2015, however, it was reported that comprehensive health literacy scores in Japan were lower than in Europe [4] and similar to the health literacy scores in six other Asian countries [13]. A comparison of health literacy in Europe and Japan showed that a higher percentage of Japanese respondents rated as “difficult” items related to evaluating information and decision-making. Health literacy is ultimately about the ability to make informed decisions and requires the identification of quality information.

One reason for Japan’s low health literacy is the lack of reliable, easy-to-understand public websites and absence of a standard information source that people can access to obtain information rapidly. One report comparing the quality of cancer information on the Internet in the United States and Japan found that US information was better because it was mainly provided by non-profit organizations and public institutions [14]. Another study in Japan found that among websites offering information about cancer treatment, the proportion of ones providing harmful information was much greater than the percentage of ones offering reliable information [15]. In Japan, there is no reliable and comprehensive website comparable to MedlinePlus (US National Library of Medicine): Japan has neither a national institute of health nor a national library of medicine. Further, few Japanese use English on a daily basis; Internet use is predominantly in Japanese, which may limit the use of useful information in English more than in Europe.

The most common health information resources for Japanese people are television and radio (77.5%), the Internet (74.6%), and newspapers (60.0%); these resources were found to be trusted by 70.5%, 55.6%, and 76.2% of respondents, respectively [16]. This high level of trust in mass media (such as newspapers and television) and low trust in the Internet is a characteristic of Japan that is not limited to health information. According to the World Values Survey, trust in newspapers and television is approximately 60%–70% in Japan compared with around 10%–40% in Western countries [17]. However, another survey showed that trust in the Internet was lowest in Japan (51%) compared with 74% overall among the 25 countries and regions surveyed [18]. Information access is changing, and many people now independently search for reliable information using the Internet. However, perhaps because it is difficult to obtain such information from the Internet, Japanese tend to rely on information from the mass media, which are less reliable than in other countries. This pattern may hinder Japanese from evaluating information for themselves and making decisions based on such information.

It is also possible that the type of education provided in Japan (from childhood onward) affects the development of information-evaluation abilities. Although Japanese are able to obtain and understand information, they are less able to evaluate it or make decisions; this is because Japanese education does not aim to develop those abilities. Only in the last few years has the ability to evaluate information and make decisions been included as a major pillar in the national curriculum guidelines for elementary through high school [19].

There are clear cultural differences in decision-making and the environment that enables it. In a comparison of Japanese and Australian university students, Japanese expressed lower self-esteem in decision-making, higher stress in decision-making, and the tendency to make decisions impatiently and incorrectly without considering other options; alternatively, they avoided decision-making rather than thinking a problem through and deciding for themselves [20]. Similarly, in a study comparing university students in Japan, Australia, the United States, New Zealand, Hong Kong, and Taiwan, Japanese had the lowest self-esteem regarding decision-making and tended to be the most avoidant and impulsive [21]. It has been pointed out that this may reflect a difference between Western culture (which promotes individual decision-making) and Japanese culture (which values group harmony). Further, a study comparing the decision-making styles of business leaders in Japan, the United States, and China found that in Japan gathering data and carefully analyzing many options was the least common style; focusing on intuition and relationships rather than data was the most common approach [22].

Similarly, regarding cultural aspects, it has been observed that Japanese have low self-esteem compared with North Americans [23]. One study reported that in contrast to North Americans’ motivations to identify what is good in themselves, Japanese appear more motivated to find areas of insufficiency [24]. However, further research is needed: there may be differences between North America and Europe even though in some areas there are few differences between those two regions.

The ability to make decisions may also be affected by whether or not there is freedom of choice. The World Values Survey contains the item “Please use the scale to indicate how much freedom of choice and control you feel you have over the way your life turns out,” which respondents rate on a scale of 1–10. In terms of average score on this item, Japan scored 81st among 83 countries and regions [17]. This may indicate that Japanese feel they have insufficient options, insufficient information to make a decision, or lack the skills to make an informed decision.

It is necessary to examine the information-evaluation and decision-making skills that are needed to make informed decisions. Many university library websites worldwide have long published methods for evaluating information resources and websites. These methods involve tests and guidelines for judging the quality of information sources. Some of the most widely used are the following: CRAP (currency, reliability, authority, and purpose) [25], developed by a university librarian, which is a helpful tool when trying to decide if a website is a credible, valid source; and CRAAP (currency, relevance, authority, accuracy, and purpose) tests [26], which is widely used as a teaching tool for college students learning how to evaluate Internet resources. Similarly developed as a checklist for students, there are five criteria known as AAOCC (accuracy, authority, objectivity, currency, and coverage) [27–29], which include and are very similar to the criteria of CRAP and CRAAP.

The five AAOCC criteria can be defined as follows. Accuracy (or reliability) is whether the information is reliable, whether it can be verified by other sources, and whether it is clear what the original sources are and whether they contain sufficient evidence. Authority means that the identity and qualifications of the author or person providing the information are clear. Objectivity (or purpose) refers to whether the information is free from bias, why it is provided, and whether it is biased for advertising or commercial purposes. Currency signifies that the accuracy of the information source depends on when the information was created and how often it is updated. It is true that many older studies are still valid today; however, it is important to have current details about when the information was provided (such as when it was published or posted) so it is possible to confirm whether it is still accurate and has not been recently adjusted. Coverage (or relevance) is the extent to which the information addresses what the user wants to know, whether it is broad in scope or specialized, and how it differs from other information. To determine this, it is necessary to compare the information with other sources and clarify the differences, rather than making a judgment based on the original information alone. Thus, it is important that people develop the skills to identify and confirm these five criteria so that information can be properly evaluated and reliable information used.

Different people make decisions in different ways. Many studies have examined the psychology of decision-making styles; several measurement scales have emerged [30, 31]. Two major decision-making styles have been identified: the rational and intuitive styles. Consistent with previous studies, Hamilton et al. defined the characteristics of the rational style as a thorough search for information and a systematic evaluation of all choices and potential alternatives; they described the characteristics of the intuitive style as applying quick decision-making that is primarily based on hunches and feelings [31]. The rational style scale includes evaluating information (such as thorough information gathering and investigating facts) and carefully considering pros and cons, or benefits and risks, to explore all options and evaluate alternatives. A rational style is needed for informed decision-making, which is the process of evaluating information (e.g., evidence and data) and options.

In the fields of business and health, individuals are required frequently to make decisions. In these areas, decision-making is a process: better decision-making demands the steps of generating options, comparing the options’ pros and cons, and then making a decision [32].

Decision-making has become integral to the research and practice of informed and shared decision-making (SDM) in the field of health [33]. According to one systematic review of SDM, its essential elements are presentation of options, discussion of pros and cons, and patient values and preferences [34]. This approach reflects the critical role in evidence-based medicine of patients’ values and preferences in decision-making [35]. Similarly, patient-centered care takes into account the preferences, needs, and values of each patient; it ensures that the patient’s values guide all clinical decisions [36]. Evidence-based medicine is an essential prerequisite of SDM [37]; Barry and Edgman-Levitan note that SDM is the pinnacle of patient-centered care [38].

A US national study to assess SDM developed a scale to assess four fundamental aspects (discussion of options, pros, cons, and preferences) that reflect the widely accepted definition of SDM [39, 40]. According to the Ottawa Decision Support Framework (a theory of decisional support for difficult decisions), the quality of decision-making requires that choices be made based on what is most important among the pros and cons of options [41]. Decision aids (DAs) are tools that support decision-making; they help people determine the pros and cons of options as well as identify their importance. Such an approach leads to effective decision-making: it amounts to recognizing that the decision is informed, value based, likely to be implemented, and will probably lead to satisfaction with the decision [42]. Recent research indicates that SDM and DA play an important role in decision-making in Japan [43–46].

The perceived difficulty of evaluating information and making decisions as health-related tasks has been included in scales measuring health literacy applied in studies comparing Europe and Asia [2, 4, 13]. The studies have assessed the ease for people to evaluate information and make decisions; however, they have not examined how the subjects did so. For individuals to assess health information, they may require specific skills for judging the reliability of diverse information using evaluation criteria. Furthermore, even if individuals possess reliable information to deal effectively with health issues, they need specific skills for implementing rational decision-making; that involves obtaining sufficient options, understanding pros and cons, and selecting the best alternative according to their values. Clarifying the association between health literacy and specific skills to evaluate information and make decisions (not necessarily confined to health information) would inform efforts to improve health literacy.

Accordingly, the objectives of the present study were to investigate the following issues using a nationwide survey in Japan. (1) When obtaining information, to what extent do individuals apply criteria for evaluating information to confirm its reliability; when making decisions, to what extent do people seek out available options and compare pros and cons based on their own values? (2) How strongly are such skills associated with health literacy and demographic characteristics? (3) What opportunities are available to acquire these skills?

## Methods

### Participants

The participants were recruited from individuals registered with a Japanese Internet research company, which had approximately 1.4 million voluntarily registered associates. We collected data from a minimum of 4,000 men and women aged 20–69 years. In January 2021, we randomly invited 22,115 potential respondents via email to participate in a cross-sectional Web-based anonymous questionnaire.

In determining potential participants, we tried to match participants’ gender, age-group, and region (we divided Japan into eight regions) according to the results of the 2015 census [47]. We accepted emailed responses from potential participants until we reached the target number for gender, age-group, and region.

### Measurements

#### Japanese version of the European health literacy survey questionnaire (HLS-EU-Q47) [4]

The survey response categories were all phrased similarly: “On a scale from very easy to very difficult, how easy would you say it is to understand why you need health screenings?”; the responses were ranked on a four-point Likert-type scale (1 = very difficult, 2 = fairly difficult, 3 = fairly easy, 4 = very easy). We also included a “don’t know/inapplicable” response option, which we coded as a missing value.

As in the original scale, we standardized health literacy scores on a metric between 0 and 50, using the formula (MEAN − 1) × (50/3) [4]. There, MEAN signified the mean of all item responses for each participant.

#### Information evaluation

On the basis of the five AAOCC criteria (accuracy, authority, objectivity, currency, and coverage) from tests and guidelines for judging the quality of information sources [26–29], we created five items to determine how frequently the participants evaluated information. In developing the questions, we referred to the following: items measuring student assessment of the reliability of information for schoolwork [48]; a guide for evaluating health-related information in a school health education text [49]; and points developed for checking health information corresponding to the five AAOCC criteria [50, 51]. In order of the five criteria, we asked respondents to rate how often they checked the following aspects of the information they accessed on the Internet, television, newspapers, magazines, or other media: (1) the source of the information; (2) the qualifications of the people and organizations providing the information; (3) whether the information advertised products or services; (4) when the information was created; and (5) how the information differed from other information. We rated responses to all items on a five-point scale (5 = always, 4 = often, 3 = sometimes, 2 = rarely, 1 = never). We calculated total and item scores. Higher scores on the scale indicated greater skill in evaluating information.

#### Decision-making process

We measured whether the essential aspects of the process of determining all available options, knowing the pros and cons of each option, comparing them based on values and preferences, and making a choice were implemented; those aspects are necessary for informed decision-making. For this purpose, we developed four items for each aspect (options, pros, cons, and values or preferences) with reference to the Shared Decision Making Process Scale [39]. Items on the scale are limited to the two options of whether to test or intervene; thus, we created items that were not limited to health decisions and had a wider range of options. We asked respondents to rate how often they implemented the following aspects when they made important decisions: (1) make sure they have all the options, (2) know the pros of each option, (3) know the cons of each option, and (4) compare the pros and cons of each option and clarify what is important to them. As with the information-evaluation items, we applied a five-point scale (5 = always, 4 = often, 3 = sometimes, 2 = rarely, 1 = never). We calculated total and item scores. Higher scores signified greater decision-making skill.

#### Learning opportunities

Immediately after answering the five information-evaluation items, participants were asked where they had acquired the skills of evaluating information: “Have you ever learned how to check information as mentioned in the previous question?” Immediately after answering the four decision-making items, participants were asked where they had acquired the skills of decision making: “Have you ever learned anywhere about how to determine the pros and cons of all options and what is important before making a choice?” The multiple-response options were as follows and respondents were asked to check whether each applied: never learned; Internet; television; newspapers and magazines; books; workplace; home; elementary school; junior high school; high school; college or university; other.

### Demographic characteristics

We examined the following demographic characteristics: gender (men, women); age-group (20–29, 30–39, 40–49, 50–59, 60–69 years); highest level of education (junior high school, high school, 2-year college, college or university, graduate school); and occupation (self-employed, managerial and administrative, professional and technical, other regular role [routine and manual], part-time, homemaker, student, unemployed).

### Statistical analysis

We examined the distribution of responses to the five information-evaluation items and four decision-making items. We calculated Pearson correlations between health literacy and total and item scores.

We confirmed the reliability and validity of the five information-evaluation items and four decision-making items. To examine internal consistency, we calculated Cronbach alphas. For construct validity, we conducted confirmatory factor analysis (CFA). In the CFA, we used the comparative fit index (CFI), root mean square error of approximation (RMSEA), and standardized root mean square residual (SRMR) as model fit indexes. A CFI value of ≥ 0.95 is generally considered to represent good model fit and ≥ 0.90 acceptable fit. RMSEA and SRMR values of < 0.05 represent good fit; a value of < 0.08 is acceptable [52].

To determine the extent to which information evaluation and decision-making independently explain health literacy, we conducted a multiple linear regression analysis with health literacy as the dependent variable and information-evaluation and decision-making scores as the independent variables. We used demographic characteristics (gender, age-group, education, and occupation) as control variables in the same analysis. Finally, we calculated the percentage of responses for each of the opportunities for learning about evaluating information and decision-making.

To determine the association between demographic characteristics and high scores on the information-evaluation and decision-making items, we conducted a multiple linear regression analysis (general linear model) with these item scores as the dependent variables; we used gender, age-group, education, and occupation as the independent variables. For comparison, we conducted the same analysis with health literacy as the dependent variable. In that analysis, we calculated the estimated marginal mean for each category to compare the mean values across categories.

Finally, we calculated the percentage of responses for each of the opportunities for learning about information evaluation and decision-making. We analyzed data using IBM SPSS Statistics (IBM Corp., Armonk, NY, USA) version 27.0 and Amos version 27.0 (IBM SPSS, Chicago, USA).

## Results

### Participant characteristics

There were 3,191 valid responses, including those with missing values of less than 20% on all health literacy items (sufficient to calculate a health literacy score in accordance with the original HLS-EU-Q47) [2]. We included data for those individuals in the analysis. Demographic characteristics and health literacy scores appear in Table 1.

**Table 1:** Characteristics of study participants

Variables	Total (3,914)	
	n	%
Gender		
Men	1,953	49.9
Women	1,961	50.1
Age-group		
20 − 29	567	14.5
30 − 39	721	18.4
40 − 49	891	22.8
50 − 59	785	20.1
60 − 69	950	24.3
Age (mean ± SD)	46.9 ± 13.6	
Highest level of education		
Junior high school	86	2.2
High school	981	25.1
2-year college	858	21.9
College or university	1,806	46.1
Graduate	183	4.7
Occupation		
Self-employed	191	4.9
Managerial and administrative	166	4.2
Professional and technical	463	11.8
Other (routine and manual)	1,367	34.9
Part-time	474	12.1
Homemaker	652	16.7
Student	131	3.3
Unemployed	470	12.0
Health literacy (mean ± SD)	27.4 ± 9.4	

*SD* standard deviation

### Distribution of responses on information evaluation and decision-making

Table 2 shows the distribution of information-evaluation and decision-making responses and their correlations with health literacy. Response distribution was similar for the five information-evaluation items: approximately 10%–15% of participants answered “always,” and around 25% responded “often.” Only the objectivity item received over 40% of “always” and “often” responses combined; approximately 30% of participants answered “always” and “often” to the other items. The average score on the five-point scale was approximately 3 (“sometimes” option). The distribution was similar for the four decision-making items. Around 10%–15% of participants answered “always”; 30%–35% responded “often”; and 40%–50% answered “always” or “often.” The average score on the five-point scale was just over 3—slightly higher than the score for the “sometimes” option.

**Table 2:** Distribution of responses for information evaluation, decision-making, and their correlations with health literacy

Variables	Responses						Correlations with health literacy	
	Always	Often	Sometimes	Rarely	Never	Mean ± SD	r	*P*
Information evaluation								
(Accuracy) I check the source of the information	12.5	24.5	27.7	17.5	17.7	3.0 ± 1.3	.24	< .001
(Authority) I check the qualifications of the people and organizations providing the information	10.5	23.3	30.0	17.9	18.3	2.9 ± 1.2	.22	< .001
(Objectivity) I check whether the information advertised products or services	14.9	28.4	27.8	15.2	13.8	3.2 ± 1.2	.23	< .001
(Currency) I check when the information was created	10.3	25.4	28.8	18.9	16.6	2.9 ± 1.2	.21	< .001
(Coverage) I check how the information differed from other information	9.7	24.6	31.0	18.7	16.0	2.9 ± 1.2	.24	< .001
Total score						14.9 ± 5.4	.26	< .001
Decision-making process								
(Options) I make sure I have all the options. available to me	8.7	31.0	34.2	19.1	6.9	3.2 ± 1.1	.24	< .001
(Pros) I know the pros of each option	11.9	35.7	32.1	16.1	4.3	3.3 ± 1.0	.28	< .001
(Cons) I know the cons of each option	12.0	34.0	32.3	17.2	4.5	3.3 ± 1.0	.27	< .001
(Values and preferences) I compare the pros and cons of each option and clarify what is important to me	14.6	35.7	31.4	13.9	4.4	3.4 ± 1.0	.30	< .001
Total score						13.2 ± 3.8	.30	< .001

*SD* standard deviation

### Correlations with health literacy

The Pearson correlation coefficients among the five information-evaluation items and health literacy ranged from 0.21 to 0.24; the highest correlations (0.24) were for the accuracy and coverage items (Table 2). The correlation between health literacy and the information-evaluation total score was 0.26 (slightly higher than the correlations for each item); all correlations were significant. Correlations between health literacy and the four decision-making items ranged from 0.24 to 0.30; the highest correlation (0.30) was for the values or preferences item. The correlation between health literacy and decision-making total score was 0.30 (greater than the correlations for each item and the same as the correlation for values or preferences); all correlations were significant. The correlation between the total information-evaluation score and the total decision-making score was 0.60 (*P* < 0.001) (not shown in Table 2).

### Reliability and validity

To confirm the reliability and validity of the total information-evaluation and decision-making scores, we first calculated Cronbach alpha coefficients, which were 0.92 and 0.93, respectively. We confirmed the construct validity of the information-evaluation and decision-making items using CFA. For the information-evaluation items, the CFI was 0.995, the RMSEA 0.067 with a 95% confidence interval (CI) of 0.055–0.081, and the SRMR.011; that indicated acceptable fit. We observed error covariance between items with similar texts (currency and objectivity items), but the CFA factor loadings were > 0.77 for all items, and we confirmed a unidimensional structure. For the decision-making items, the CFI was 0.998, the RMSEA 0.061 with a 95% CI of 0.043–0.083, and the SRMR.007; that indicated acceptable fit. All the CFA factor loadings were > 0.76 for all items, and we confirmed a unidimensional structure.

### Multiple linear regression analysis of health literacy

Table 3 shows the results of the multiple linear regression analysis with health literacy as the dependent variable and the information-evaluation and decision-making scores as the independent variables. The standardized regression (beta) coefficients were 0.13 for information evaluation and 0.22 for decision-making; both were significant. When we used demographic characteristics as control variables, the coefficients were 0.15 and 0.22, respectively; both were significant.

**Table 3:** Multiple linear regression analysis of health literacy

	Health literacy			Health literacy controlling for demographic variables ^a^		
Independent variables	Beta ^b^	*t*	*P*	Beta	*t*	*P*
Information evaluation	.13	7.01	< .001	.15	8.05	< .001
Decision-making process	.22	12.01	< .001	.22	11.95	< .001
R^2^		.10			.14	
Adjusted R^2^		.10			.13	
F		221.05			33.95	
*P*		< .001			< .001	

^a^ Gender, age-group, education, occupation

^b^ Standardized regression coefficients

### Multiple linear regression analysis of information evaluation and decision-making

Table 4 presents the results of the multiple linear regression analysis with the information-evaluation and decision-making item scores as the dependent variables and demographic characteristics as the independent variables. To compare these results, we conducted a similar analysis with health literacy as the dependent variable. Women scored slightly higher on decision-making. Participants in their 20 s scored slightly higher than those in other age-groups on both variables. Participants with a graduate education scored highest; they were followed by individuals with a college or university education (for information evaluation). College students scored highest on both variables; they were followed by self-employed participants and those in managerial or administrative positions (for decision-making).

**Table 4:** Multiple linear regression analyses of information evaluation, decision-making process, and health literacy

Variables	Information evaluation			Decision-making process			Health literacy		
	EMM (95% CI)	*F*-test	*P*	EMM (95% CI)	*F*-test	*P*	EMM (95% CI)	*F*-test	*P*
Gender		0.15	.7		5.44	.02		20.48	< .001
Men	15.3 (14.9, 15.7)			13.5 (13.2, 13.8)			26.7 (26.0, 27.3)		
Women	15.2 (14.8, 15.6)			13.8 (13.6, 14.1)			28.3 (27.6, 29.0)		
Age-group		5.19	< .001		4.14	.002		15.60	< .001
20 − 29	16.2 (15.7, 16.8)			14.2 (13.8, 14.6)			27.0 (26.1, 27.9)		
30 − 39	15.4 (14.9, 15.8)			13.7 (13.4, 14.1)			26.1 (25.2, 26.9)		
40 − 49	14.9 (14.5, 15.4)			13.5 (13.2, 13.8)			26.9 (26.2, 27.7)		
50 − 59	14.8 (14.3, 15.3)			13.3 (13.0, 13.6)			27.8 (26.9, 28.6)		
60 − 69	15.1 (14.6, 15.6)			13.6 (13.3, 13.9)			29.5 (28.7, 30.3)		
Highest level of education		17.98	< .001		10.69	< .001		4.30	.002
Junior high school	14.6 (13.5, 15.8)			13.5 (12.7, 14.3)			25.6 (23.6, 27.6)		
High school	14.2 (13.9, 14.6)			13.0 (12.8, 13.3)			27.3 (26.7, 27.9)		
2-year college	14.7 (14.3, 15.1)			13.3 (13.0, 13.6)			27.0 (26.3, 27.7)		
College or university	15.7 (15.4, 16.0)			13.7 (13.5, 13.9)			27.5 (27.0, 28.0)		
Graduate	17.1 (16.3, 17.9)			14.8 (14.2, 15.3)			29.9 (28.5, 31.3)		
Occupation		2.86	.006		3.63	< .001		1.23	.283
Self-employed	15.5 (14.6, 16.3)			14.3 (13.7, 14.9)			28.5 (27.1, 29.9)		
Managerial and administrative	15.5 (14.6, 16.4)			14.1 (13.5, 14.7)			28.4 (26.8, 29.9)		
Professional and technical	15.7 (15.2, 16.3)			13.5 (13.1, 13.9)			27.0 (26.1, 28.0)		
Other (routine and manual)	15.1 (14.7, 15.5)			13.4 (13.2, 13.7)			27.0 (26.4, 27.7)		
Part-time	14.8 (14.2, 15.3)			13.3 (13.0, 13.7)			27.7 (26.8, 28.7)		
Homemaker	14.7 (14.1, 15.2)			13.2 (12.8, 13.6)			27.4 (26.5, 28.3)		
Student	16.4 (15.3, 17.4)			14.3 (13.6, 15.0)			26.7 (24.9, 28.5)		
Unemployed	14.6 (14.1, 15.2)			13.1 (12.7, 13.5)			26.9 (26.0, 27.8)		

*CI* confidence interval, *EMM* estimated marginal mean

The analysis of health literacy identified significant differences for gender, age, and education; there were higher scores for women than for men, higher scores for older age-groups (except for individuals in their 20 s), and higher scores for graduates. These results conform with those of Nakayama et al. [4]. The health literacy results were similar to those for information evaluation and decision-making: scores were slightly higher for participants in their 20 s and higher for graduates.

### Learning opportunities

Table 5 displays the results for the multiple-choice question asking where respondents learned about information evaluation and decision-making. Responses to both were similar: the most frequent answer was “never learned” (> 40%). The most frequent means of learning was the Internet (approximately 40%); it was followed by television (around 30%), newspapers and magazines (about 15%), books (just over 10%), and other sources (< 10%).

**Table 5:** Multiple responses by participants as to where they learned information evaluation and decision-making skills (if none applied, they answered “Never learned”) (%)

	Information evaluation	Decision-making process
Never learned	42.4	44.3
Internet	39.9	38.8
TV	32.2	29.0
Newspapers and magazines	15.3	15.0
Books	12.3	13.0
College or university	6.5	6.0
Workplace	5.7	5.8
Home	4.1	5.7
Elementary, junior high, or high school	4.9	4.4
Others	4.7	4.0

## Discussion

This study found that information-evaluation and decision-making skills are associated with health literacy. These skills may lead to interindividual differences in health; thus, it is necessary to provide learning opportunities to eliminate such differences. However, health literacy is a relational concept; accordingly, both individual skills and interactions between people and their environment need to be enhanced [53]. It is necessary to create an environment in which all individuals can easily receive support at any time; in that way, they can obtain reliable health information and engage in decision-making. However, we did not find that the associations between health literacy and individual skills were very strong. That may have been because our participants were not necessarily using or attempting to apply the skills in the tasks measured by our health literacy scale. Future research should clarify appropriate methods for evaluating information and making decisions with respect to the tasks on health literacy scales.

We found that only approximately 30% of participants always or often evaluated health information; 70% sometimes (or less frequently) evaluated this information. Such a pattern is good if individuals receive high-quality information but potentially risky if they do not. Many people need to increase their ability to identify useful information; this is particularly important for health information (e.g., information about cancer) because much of the available information is unreliable. It is necessary for information providers to offer reliable, easily understandable information through the channels of the target audience. For example, efforts to achieve this in the United States have resulted in the Health Literacy Online [54] website, which covers information on how to create an easy-to-understand health information portal. The US Centers for Disease Control and Prevention offers a guide to creating easily understandable materials [55]. MedlinePlus has an Easy-to-Read section [56], which provides easily understandable materials in an A–Z format. The Health Literacy Universal Precautions Toolkit developed by the US Agency for Healthcare Research and Quality [57] is an evidence-based guide to help health-care providers provide understandable information for patients. Equivalent sources have not been fully developed in Japan, and there is a need for further research to provide such information.

In this study, both simple correlations and multivariate regression analysis showed that decision-making skills correlated slightly more highly with health literacy than did information-evaluation skills. In our multivariate analysis, the association between decision-making skills and health literacy was greater after controlling for information-evaluation skills. This finding indicates that even if information is properly evaluated, the skill in taking part in decision-making is important in health literacy, which is the ability to make informed decisions. This means that the skill to engage in decision-making is an important part of health literacy.

If decision-making is conceptualized as a skill, it is possible to assess whether individuals possess this skill or not—and whether they require support. In addition, identifying the processes people engage in during decision-making enables such processes to be assessed and evaluated; they can be shared with others to gain understanding and cooperation. It could be useful to make decisions in consultation with family members and others if the outcome of those decisions affects them. However, fewer than half of our respondents always or often engaged in decision-making with such individuals; this finding suggests that many people lack the skills for (or are unable to perform) informed decision-making. In addition to enhancing decision-making, SDM and the associated DA tool need to be developed and disseminated to support that process.

We found that participants in their 20 s, those who had completed graduate school, and college students scored higher in decision-making; however, they did so by only approximately 1 percentage point. Postgraduates and regular college students comprised less than 5% of the sample. We observed generally little difference between participants of different ages and educational backgrounds. In terms of occupation, participants in the self-employed and managerial and administrative categories scored slightly higher; that was probably because decision-making is a necessary skill for workplace leaders. However, we observed few demographic differences in the overall results.

This study highlighted the lack of learning opportunities. Only slightly more than half of respondents had ever learned about information evaluation or decision-making. Those who had learned about these skills tended to do so through their own efforts, using the Internet, television, or books; they had received limited opportunities to learn in school or at work. Attempts to increase health literacy must take into account individual opportunities and motivation to learn about information evaluation and decision-making. However, some of our “never learned” responses may have included cases where participants were unable to recall where they had learned something but had learned experientially without realizing. To ensure that such skills are acquired, a formal learning program that is memorable may be requisite.

As noted above, research had demonstrated that Japanese tend not to make informed decisions independently [20–22]. Thus, an important question is whether Japanese find value in making such independent decisions. Worldwide, the main factors that improve happiness and life satisfaction are health status, household financial satisfaction, and freedom of choice [58]. One of the ethical principles underlying SDM is that self-determination is a desirable goal and plays an important role in individual well-being [59]. It remains to be established whether Japanese consider such characteristics desirable.

A nationwide survey among 20,000 Japanese participants identified self-determination as the second-most important determinant of happiness after health and relationships—more so than income and education [60]. This is because self-determination leads to greater motivation and satisfaction with a person’s chosen actions; that ultimately leads to a greater sense of well-being [59]. Regarding SDM, one Japanese survey found that when deciding how to treat a relatively serious illness, participants were more likely to want to decide for themselves than to let a doctor make decisions [61]. Another study identified a strong relationship among SDM, effective decision-making, and satisfaction with physician explanations [46]. Thus, although many Japanese desire self-determination, they may have limited opportunities to learn the relevant skills or may not receive the appropriate support. Such a situation makes it difficult for individuals to express themselves, and it may be assumed that individuals in that situation have less freedom of choice [17]. It is also possible that many people’s desire for self-determination—and the idea that self-determination leads to happiness—is not widely disseminated. This point may need to be addressed to improve health literacy.

It is necessary to examine the strategies that could be used to promote decision-making skills and improve health literacy. In the Ask 3 Questions (a health literacy campaign to promote SDM), participants were asked the following: What are my options?; What are the pros and cons for each option?; and What support is available to help me make a decision? In the AskShareKnow framework, the first two questions are almost identical to the Ask 3 Questions items; the third is “How likely are each of those benefits and harms to happen to me?” In the Choosing Wisely initiative, which promotes better decision-making in health-care, four questions are recommended (BRAN—from the following key words): what are the benefits?; what are the risks?; what are the alternatives?; and what if I do nothing? These initiatives all contain a question about options and pros and cons; however, they do not include items about which pros and cons are important. A question such as “What support is available if I don’t know which pros and cons are important?” would be useful. Findings from recent studies linking health literacy and SDM using the AskShareKnow framework are useful [62]; however, intervention studies applying such questions are required.

The present study has several limitations. It is possible there was some sample selection bias. Our study may have been skewed toward a high level of Internet literacy because it was a Web-based survey. We based recruitment of respondents on self-selection from a group of individuals who had previously expressed a desire to participate in research projects. The responses were limited to approximately the first 4,000 people, which may have included only those who were most active on the Internet (e.g., ones who frequently checked e-mail).

Users familiar with the Internet and social media may find it easier to obtain health information; however, they may become confused by the large amounts of often contradictory, inaccurate, or poor-quality health information available. Evidence suggests that Internet literacy is not the only factor determining whether people access health information electronically [63]. Our results indicate that even individuals with moderate Internet literacy also need information-evaluation and decision-making skills to enhance their health literacy. Future studies should identify whether the skills measured in this study are sufficient to identify the growing amount of unreliable health information on the Internet and—if not—what skills are lacking.

We created five items to determine how frequently the participants evaluated information. However, those were just representative items; many alternative, more detailed items could have been chosen (e.g., “What is the affiliation of the source of the information?”). However, rather than covering a wide range of content, our goal was to identify the core aspects of information evaluation associated with health literacy. Our five items were all associated with health literacy; thus, it would be beneficial to consider what other checks could be created for those five criteria when developing learning content and evaluation scales that cover such content. It is also necessary to make the evaluation criteria easier to remember (e.g., by using fewer abbreviations, such as CRAP, CRAAP, and AAOCC) if dissemination is a priority.

These issues also apply to the four decision-making items. For example, decision-making includes clarification of the problem before checking the options as well as action and evaluation after the decision is made. However, because we used SDM as a reference, we focused on the process required to make a decision, assuming that the problem was already apparent. Our aim was not to create a scale to cover all skills needed in decision-making; it was to determine whether specific key points were related to health literacy. All four items were related to health literacy; however, it would be advisable to supplement those with other items on decision-making when developing more comprehensive learning content and measures. Regarding the memorability of evaluation criteria, the decision-making process of GOFER (goals—survey values and objectives; options—consider a wide range of alternative actions; facts—search for information; effects—weigh the positive and negative consequences of the options; review—plan how to implement the options) was developed to teach adolescents to make better decisions. Accordingly, the use of memorable abbreviations can sometimes be effective [64].

In terms of scale development, Woudstra et al. developed a context-based assessment of health literacy and interventions to support informed decision-making with respect to colorectal cancer screening [65]. They suggested that health literacy skills are needed for informed decision-making according to the stages of that process. Among those stages, that of “structure decision options and outcomes” requires the skills of understanding information about possible options and outcomes and understanding merits and demerits. In the “evaluate options” stage, the skill of assessing pros and cons for personal relevance is required. These skills are covered by the items applied in the present study, but they also address the stages before and after decision-making: they measure the skills needed in the eight stages of decision-making, from receiving information about screening and undergoing screening to interpreting the screening results.

Decision-making is not always performed rationally, and the various biases associated with it (e.g., framing effects) are often not apparent. Therefore, it is desirable to include such factors in strategies to improve health literacy. Knowing what biases exist and being able to avoid them may be associated with health literacy; however, that is an issue for future research.

Another limitation of this study is that we used cross-sectional data, so we were unable to draw any firm conclusions about causal effects among the variables. This was an anonymous online survey, but the measures were self-reported data and may have been vulnerable to social desirability bias: respondents may have felt that their responses should have reflected what they ought to have been doing rather than what they actually did.

## Conclusions

In this study, we examined the skills necessary to make informed decisions about health; the skills included that of evaluating the reliability of information from various media sources (not necessarily limited to health information) and decision-making skills about understanding available options, grasping the pros and cons of each option, comparing them, and choosing the option that matched the respondent’s values. We conducted this Web-based cross-sectional nationwide survey to measure the extent to which those skills were being implemented and to examine their association with health literacy. We found that both information-evaluation and decision-making skills were associated with such literacy. However, such skills are not sufficiently widespread in Japan because there are few opportunities to learn them. Additional research is required to raise awareness of the need to acquire those skills to improve health literacy and to provide learning methods and opportunities. It is also necessary to create an environment in which all individuals can easily receive support at any time so that they can make decisions based on reliable information.

## Supplementary Information

**Additional file 1.** 

## Data Availability

Requests for the study materials and dataset used to support the conclusions of this article should be directed to the corresponding author.

## Abbreviations

AAOCC: Accuracy, authority, objectivity, currency, and coverage
CFA: Confirmatory factor analysis
CFI: Comparative fit index
CRAAP: Currency, relevance, authority, accuracy, and purpose
CRAP: Currency, reliability, authority, and purpose
DA: Decision aids
HLS-EU: European Health Literacy Survey
RMSEA: Root mean square error of approximation
SDM: Shared decision-making
SRMR: Standardized root mean square residual
